# Selected acute phase CSF factors in ischemic stroke: findings and prognostic value

**DOI:** 10.1186/1471-2377-11-41

**Published:** 2011-03-30

**Authors:** Maia Beridze, Tamar Sanikidze, Roman Shakarishvili1, Nino Intskirveli, Natan M Bornstein

**Affiliations:** 1 Department of Neuromedicine, Tbilisi State Medical University, Tbilisi, Georgia; 2 Department of Biophysics and Biochemistry, Tbilisi State Medical University, Tbilisi, Georgia; 3 Department of Neurology, Tel-Aviv Sourasky Medical Center, Sackler Faculty of Medicine, Tel-Aviv University, Tel-Aviv, Israel

**Keywords:** brain, ischemia, inflammation, oxidative stress

## Abstract

**Background:**

Study aimed at investigation of pathogenic role and prognostic value of several selected cerebrospinal fluid acute phase factors that can reflect the severity of ischemic brain damage.

**Methods:**

Ninety five acute ischemic stroke patients were investigated. Ischemic region visualized at the twenty fourth hour by conventional Magnetic Resonance Imaging. Stroke severity evaluated by National Institute Health Stroke Scale. One month outcome of disease was assessed by Barthel Index. Cerebrospinal fluid was taken at the sixth hour of stroke onset. CSF pro- and anti-inflammatory cytokines were studied by Enzyme Linked Immunosorbent Assay. Nitric Oxide and Lipoperoxide radical were measured by Electron Paramagnetic Resonance. CSF Nitrate levels were detected using the Griess reagent. Statistics performed by SPSS-11.0.

****Results**:**

At the sixth hour of stroke onset, cerebrospinal fluid cytokine levels were elevated in patients against controls. Severe stroke patients had increased interleukin-6 content compared to less severe strokes (P < 0.05). Cerebrospinal fluid Electron Paramagnetic Resonance signal of nitric oxide was increased in patients against controls. Severe stroke group had an elevated Electron Paramagnetic Resonance signal of lipoperoxiradical compared to less severe stroke. Cerebrospinal fluid nitrate levels in less severe stroke patients were higher than those for severe stroke and control. Positive correlation was established between the initial interleukin-6 content and ischemic lesion size as well as with National Institute Health Stroke Scale score on the seventh day. Initial interleukin-6 and nitrate levels in cerebrospinal fluid found to be significant for functional outcome of stroke at one month.

****Conclusion**:**

According to present study the cerebrospinal fluid contents of interleukin-6 and nitrates seem to be the most reliable prognostic factors in acute phase of ischemic stroke.

## Background

Modern concepts of acute cerebral ischemia highlight the role of neurovascular units and emphasize the importance of integrative tissue responses that result from dynamic interactions of endothelial cells, vascular sooth muscles, matrix elements, astroglia, microglia and neurons. By means of inflammatory stimuli and excitoxicity, such interactions create many sources of free toxic radicals and reactive oxygen spices [1].

In physiological conditions, endogenous protective mechanisms stabilize the levels of free oxygen radicals and reduce the oxidative/nitrosative stress reaction. In conditions of severe ischemia, rapid failure of the antioxidation protective system assists in the accumulation of arachidonic acid, prostaglandins, superoxide anion, NO and other aggressive substrates, which lead to the destabilization of cellular membranes, further damage of the blood-brain barrier, disintegration of DNA and, ultimately, to neuronal death [2]. Current therapeutic options for acute brain ischemia are concentrated on thrombolytic treatment, but this therapy is restricted to a small proportion of patients [3]. There is a need to devise a more effective protective and repair strategy and cellular treatment. The precise neurochemical alterations that take place in human's stroke still remain to be clarified and the cerebrospinal fluid (CSF) is the closest environment reflecting the immediate immunobiochemical changes in the ischemic brain tissue. The purpose of the present research was to investigate the importance and prognostic value of several selected CSF acute phase factors that are known to reflect the severity of ischemic brain damage.

## Methods

A total of 95 acute ischemic stroke patients, 54 female and 41 male, aged 45-70 years, who had been admitted to the Neurological Clinic of Tbilisi State Medical University during 2005-2009 were studied. Exclusion criteria comprised acute inflammatory and autoimmune disorders, severe somatic pathology, cancer, coma, space occupying hemispheric and cerebellar ischemic strokes. Patients and controls that used the anti-inflammatory medications for the 1-year period prior to the current research were also excluded from the study. Upon admission, a conventional CT scan was performed to exclude a brain hemorrhage. The control group consisted of 25 age-matched patients with vertebral discopathies, who showed no signs of cerebrovascular pathology. The local ethics committee approved the protocol, and informed consent was obtained from all participants or their surrogates

The etiology of stroke was classified according to TOAST criteria [4]. Medical records were retrospectively reviewed for selected non-modifiable and modifiable risk factors of stroke, including age, sex, inheritance, history of a transient ischemic attack (TIA) or a previous stroke, hypertension, atherosclerosis, atrial fibrillation, diabetes mellitus, smoking, alcohol abuse, acute infections 1-2 months before stroke, and psychological stress. The latter was ascertained by self-report in refuges from problematic regions of Georgia. Psychological stress was of interest to determine whether it can influence the inflammatory markers or functional outcome of the disease [5,6].

Upon admission, body temperature and blood pressure were recorded. Next, chemistry, basic hematology, chest X-rays, and electrocardiography were performed. Selected patients did not display marked hyperthermia or infective complications. Patients were managed according to evidence-based stroke guidelines. Thrombolytics, hemodilution, corticoids, and Nimodipine were not applied. Patients were strictly controlled and administered for hypo- and hyperglycemia and hypertension. Antiplatelet drugs were used in atherothrombotic and lacunar infarcts and anticoagulants -in suspected cardioembolic infarcts when the initial CT or magnetic resonance imaging (MRI) scans did not show a large cerebral lesion.

The ischemic region was visualized at the twenty-fourth hour from stroke onset by conventional magnetic resonance imaging (MRI; magnet operating 1,5 Tesla, Vision, Siemens) that provided axial T1, T2 images with a slice thickness of 5 mm. Radiologists who were blinded to study protocol defined the whole lesion volume by multiplying the area of focal hyperintensity by interslice gap. Stroke severity at admission and on the seventh day was evaluated by the National Institute Health Stroke Scale (NIHSS). Patients were divided in two groups: Group 1 included the patients with severe stroke (NIHSS ≥15) and Group 2 included the patients with mild and moderate stroke (NIHSS <15) [7]. Functional outcome was evaluated by Barthel Index at 1 month of stroke onset (BI) [8,9].

### Immunological Assay

For special laboratory investigations, 10 ml of CSF was taken from patients and controls at the sixth hour from stroke onset. Eight-ml CSF samples were frozen at -20°C for further assays, and the other 2 ml of CSF were frozen in liquid nitrogen for electron paramagnet resonance (EPR) study. CSF (5 ml) levels of the pro-inflammatory cytokines: interleukin-1β(IL-1β), interleukin-6(IL-6), tumor necrosis factor-α(TNF-α) and the anti-inflammatory cytokine interleukin-10 (IL-10) were detected by enzyme-linked immunosorbent assay (ELISA), by application of ELISA- RIDER. The relationship between optical density and cytokine concentrations was defined using the standard curve according to kit instructions (Bender Med systems Diagnostics, Vienna, Austria).

### Electron Paramagnetic Resonance (EPR) Study

Nitric oxide (NO) and lipoperoxide radical (LOO-) were measured by EPR spin labeling (radiospectrophotometer ESR-231 (X- band), with a modulation frequency of 50 KHZ and a TM-110 cavity). Diethyldithiocarbamic acid (DETC) (Sigma) was used as an NO trap. CSF samples were incubated with Fe2+(DETC)2 stock solution. The 0.8 mM Fe2+ (DETC) colloid solution formed was yellow-brown in color and was used immediately after preparation. EPR specters of NO-Fe2+ (DETC)2 complexes were defined at the temperature of liquid nitrogen on a microwave power of 20 mVt. The amount of detected NO was determined from the calibration curve for integral intensity of the EPR signal of NOFe2+ (DETC)2, prepared at various concentrations (1-20 μM) of the NO-donor MAHMANONOate [10]. LOO- trap, α-phenil-tert-butilitron (PBN) (SIGMA) was used at a dosage of 50 ml/0.5 ml CSF. EPR specters of LOO- were defined at room temperature on a microwave power of 20 mVt. EPR signals of LOO- were measured in arbitrary units (a.u.) (signal intensity in millimeters represented milliliters of CSF matter) [11].

### Biochemical Assay

For nitrate (NO2) detection, 3 ml CSF samples were processed by 20% Griess reagent. We used a CF-46 LOMO spectrophotometer for colorimetric detection. Optical density was detected on a 540nm wavelength. NaNO2 (5 μmol/L) was used for drawing the calibrating curve [12].

### Statistics

The obtained data were analyzed using SPSS 11.0 computer software. Normally distributed continuous variables were compared with repeated measure ANOVA, and the Kruskall-Wallis test compared abnormally distributed variables. The *χ*^2^-test was used to assess associations among categorical variables. The effect of acetyl salicylic acid (aspirin) and HMG-CoA reductase inhibitors (statins) was separated by partial correlation analysis. Spearman rank correlation and multiple logistic regression (forward stepwise conditional model) was used when all acute phase factors and stroke risk factors were entered into the model. Aspirin and Statins included in regression analysis as categorical covariate variables. The Hosmer and Lemeshow test was used to assess the goodness of fit of each model.

## Results

The main characteristics of each clinical group are presented (Table 1). At the sixth hour from stroke onset, the CSF proinflammatory cytokine levels in both study groups were elevated compared to the control (*P *< 0.05). There was no significant difference in IL-1β and TNF-α contents between the two groups, while Group 1 had significantly increased IL-6 contents compared with Group 2 (*P *< 0.05). The anti-inflammatory cytokine IL-10 levels were not significantly elevated in the two study groups compared to the control (*P *< 0.07), although there was a trend towards an increase in Group 2 (Table 2).

**Table 1:** Main Characteristics of ischemic stroke patients of Group 1 and 2.

Characteristics	Group 1 (n = 44)	Group 2 (n = 51)
**Male (%)**	65	60

**Age (years)**	57.3 (12.2)	57.1 (12.9)

Inheritance (%) **	37.8	14.7

History of TIA or previous stroke (%)**	18.9	6.4

**History of myocardial infarction (%)**	4.2	2.1

**SBP, mm Hg**	160.6(25.5)	165.8 (30.8)

**DBP, mm Hg**	90.2 (15.4)	92.4(14.6)

**Body mass index, kg/m^2^**	27.2 (5.8)	28.4 (2.6)

**Total/HDL cholesterol, ratio**	4.3 (1.2)	3.9(1.4)

**Triglycerides, mg/dL**	136 (69)	131 (72)

**C reactive protein, mg/L**	3.9 (1.2)	3.1(0.6)

Atrial fibrillation (%) *	17.8	12.6

**Serum glucose, mg/dL**	157.1 (58.6)	156.2(59.9)

**Smoking (%)**	32.6	35.7

**Alcohol abuse (%)**	4.2	5.3

**Acute infections 1-2 months before stroke (%)**	3.1	4.2

Psychological stress (%)*	17.8	11.5

**Aspirin usage in current stroke %**	32.6	34.8

**HMG-CoA reductase inhibitors in current stroke (%)**	10.5	8.4

**Fibrinogen, mg/dL**	422.1 (100.2)	410.4 (106.8)

**Leukocyte count, ×10^9^/L**	8.8 (2.2)	7.9 (2.6)

**Temperature at admission (C°)**	37.0 (1.4)	36.9(1.7)

Ischemic lesion volume (cm3)*	88.8 (11.7)	41.7 (9.6)

NIHSS score at admission **	20.2 (4.1)	8.6 (4.9)

NIHSS score on 7^th ^day **	18.5 (3.2)	7.3 (3.5)

**Infarct topography**		

Cortical (%)**	30.5	17.8

Subcortical (%) **	16.8	35.7

**Stroke etiology**		

**Large-artery atherosclerosis (%)**	12.9	8.4

Cardioembolism (%)**	28.4	13.6

Small vessel occlusion (lacunar) (%)**	0	25.2

**Other determined etiology (%)**	3.1	2.1

**Undetermined etiology (%)**	3.1	4.2

Numbers represent mean (SD) or percentage as appropriate. NIHSS values represent median (interquartil range).

*P < 0.05 ** *P *< 0.001

**Table 2:** Comparison of selected CSF acute phase factors in clinical groups and control at the sixth hour from stroke onset.

	IL-1β (pg/ml)	IL-6 (pg/ml)	TNF-α (pg/ml)	IL-10 (pg/ml)	NO_2_ (μmol/L)	NO (μmol/L)	LOO^.^ (a.u)
Control	*0.95 ± 0.02	*1.9 ± 0.09	*14 ± 2.3	3.6 ± 1.2	*102 ± 15.9	*2.78 ± 0.16	0
Group I	34.14 ± 4.7	*58 ± 4.6	44 ± 5.4	5.9 ± 1.4	*121 ± 4.56	33.8 ± 7.1	*18 ± 4.1
Group II	30.4 ± 7.3	*21.8 ± 4.4	39.4 ± 9.4	7.1 ± 1.2	*158 ± 3.13	30.18 ± 6.8	*34 ± 7.1

Data are expressed as means (SD).

**Figure 1:**
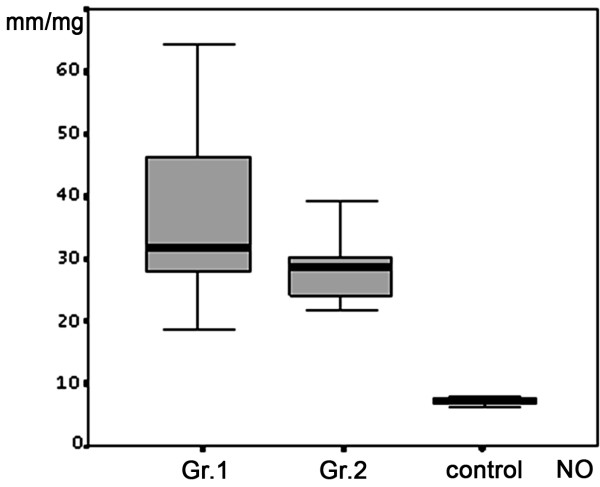
**Comparison of EPR signal intensity of CSF free NO between the study groups and control**. Box plots represent mean values (SD). *P *< 0.05 between the Group1, Group 2 and control.

**Figure 2:**
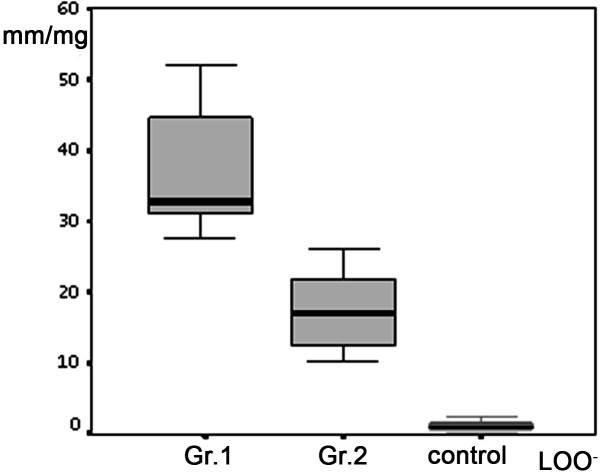
**Comparison of EPR signal intensity of CSF free LOO- between the study groups and control**. Box plots represent mean values (SD). *P *< 0.05 between the study groups and control. *P *< 0.50 between the Group 1 and Group 2.

**Figure 3:**
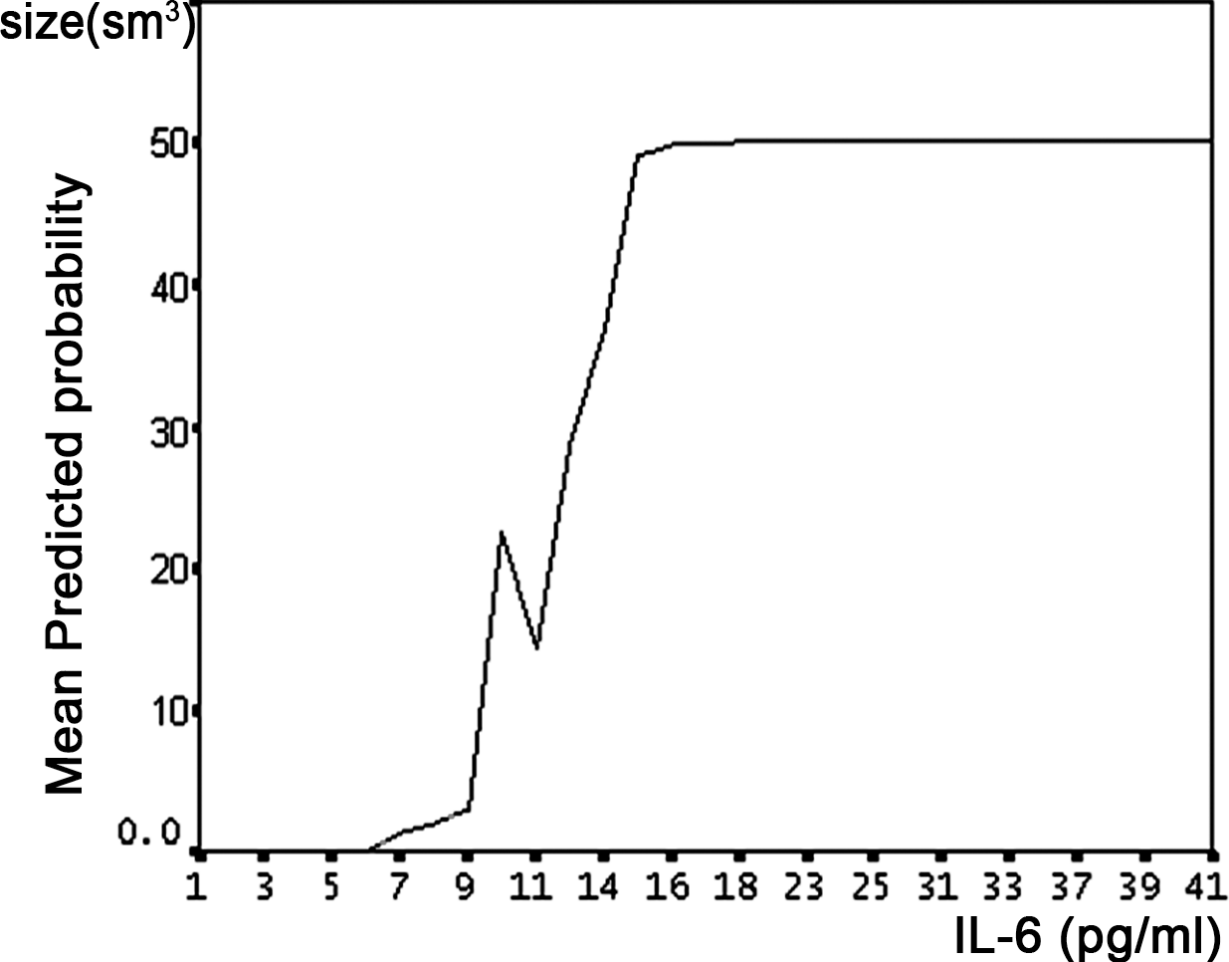
**Relation of initial CSF IL-6 content with ischemic lesion size 24 hours from stroke**. After all acute phase factors and risk factors entered into the stepwise model of multivariate logistic regression only initial CSF IL-6 gained significance and positively correlated with mean predicted probability of brain ischemic lesion size (r = +0.34; *P *< 0.05).

The EPR signal intensity of NO was increased in Groups 1 and 2 compared to the Control (*P *< 0.05), but not between the study groups (*P *< 0.50) (Figure 1). The EPR signal intensity of LOO- in Groups 1 and 2 was significantly increased compared to the control (*P *< 0.05), and Group 1 had an elevated EPR signal intensity of LOO- compared to Group 2 (*P *< 0.05) (Figure 2). The NO2 levels for Group 2 were higher than those for Group 1 and the controls (Table 2). At the twenty-fourth hour from stroke onset, the mean ischemic lesion size (cm**^3^**) for Group 1 was significantly increased as compared to Group 2 (Table 1). There was a significant positive correlation between the initial IL-6 contents and ischemic lesion size (r = +0.34; *P *< 0.05). The effect of Aspirin and Statins did not change the zero ordered correlations between study variables. Multivariate logistic regression analysis established a level of significance of IL-6 contents toward the mean predicted probability of ischemic lesion size at the twenty-fourth hour from stroke onset, after all the acute phase factors and risk factors that we examined were entered into the model (Figure 3). A positive correlation was established between the initial IL-6 CSF levels and the NIHSS scores on the seventh day of stroke (r = +0.52; *P *< 0.05). There was no significant correlation between the CSF inflammatory markers and cortical or sub-cortical ischemic lesion sites. Researched factors found to be dependent on each other once all of them were included in stepwise logistic regression analysis toward the stroke functional outcome. Only the initial IL-6 and NO_2 _levels retained significance for functional outcome of stroke at one month (Table 3), and cardiogenic strokes showed borderline significance (*P *= 0.057). Effect and interactions of Aspirin and Statins were not significant in the given model. There was a negative correlation between the initial IL-6 levels and functional outcome (BI) of stroke at 1 month (r = - 0.45: *P *< 0.05).

**Table 3:** Relationship between selected acute phase factors and functional outcome of stroke at one month.

CSF Acute phase factors	Regression Coefficient	Standard Error	*P *Value
IL-6	3.08	0.014	0.031

NO_2_	8.06	2.14	0.001

(Multivariate logistic regression analysis)

Only variables statistically significant in the final model are shown.

## Discussion

It is believed that the first local, glial immune response of the brain tissue to acute ischemia is the connection of CD4 T lymphocytes with astrocytes. Activated CD4 cells produce γ-interferon, which stimulates astrocytes to express HLA-II class antigens and to produce IL-1β. The latter stimulates phagocyte activity in glial tissue and induces production of IL-6 and TNF-α, the cytokines of initial local inflammatory reactions that trigger the subsequent development of the pro- and anti-inflammatory cytokine cascade [13].

Experimental and clinical studies have demonstrated that the high CSF and blood concentrations of proinflammatory cytokines appear to reach a peak response by 24-48 hours from stroke onset [14]. The present study found elevated IL-1β, IL-6 and TNF-α level in CSF at the sixth hour from ischemic stroke onset. Previously published studies have demonstrated that elevated CSF and plasma levels of IL-1β correlate with larger brain infarcts and worse functional outcome [15,16]. The present study did not show any significant group differences in the IL-1β and TNF-α CSF levels at six hours of ischemic stroke. However, the absolute number of these cytokines was elevated in the severe stroke group, suggesting that they are of the first proinflammatory response and may trigger the subsequent proinflammatory cascade.

The significant group differences in the initial IL-6 levels and the positive correlation with the size of the ischemic region support earlier experimental and clinical studies that revealed a correlation between increased initial CSF and plasma IL-6 levels, on one hand, and larger brain lesion volume and poor outcome on the other [17]. According to a number of experimental studies, most animals retain high blood IL-6 levels during the one-year period after ischemic brain injury. IL-6 has a mitogenic effect on astrocytes and induces reactive gliosis in later stages of brain ischemia [18]. Thus, according to the present research, IL-6 tends to reflect the severity of the stroke even at six hours post-injury and may play a key role in inflammatory damage caused by ischemia.

Previous experimental and clinical studies have shown that a high initial expression of TNF-α is connected with larger brain infarcts, and TNF-α knockout animals have larger infarcts and decreased neuronal survival [19]. Expression of TNF-α during the critical period of a stroke may restrict aggressive immune responses because the TNF signaling pathway involving CD95-CD95L (ligand) interactions is considered to be the controlling mechanism of T cell expansion during the immune response [20].

As mentioned above, the CSF levels of IL-1 β and TNF-α were found to be increased, but not significantly, in severe stroke patients at the time point examined in this study but might become significant at later stages of stroke. Because we could not find a significant correlation with these CSF markers and infarct size six hours after stroke onset, we hypothesize that CSF IL-6 could rapidly and specifically react in areas of ischemic damage with increased activity at later time points and retaining high meanings for a longer period. However, further studies are necessary to confirm this hypothesis. The anti-inflammatory cytokine IL-10 reaches its peak expression between 2-7 days after stroke onset and limits the production of proinflammatory agents through negative feedback mechanisms [21]. We could not confirm previous studies where initial IL-10 levels in blood were linked with infarct topography [22], which may be due to the relatively short time for IL-10 expression (6 hours). In keeping with previous studies, this study also connected the borderline significance of cardioembolic stroke to poor outcomes in selected patients, which might be explained by the likely association of elevated cardiac inflammatory markers in this stroke subtype [23].

In conjunction with the release of pro-inflammatory agents and glutamate toxicity, local inflammation, as described above, results in free radical pathology that directly and indirectly damages neurons by destabilizing cell membranes, disintegrating DNA, and switching the pathways of delayed neuronal death [24]. In the present study, the high EPR signal intensity of free CSF NO may be caused primarily by deregulation of the neuronal form of NO-synthase. However, NO produced by inducible and endothelial forms of NO-synthase can also pass the damaged blood-brain barrier and accumulate in CSF. The toxic effects of NO in the ischemic brain depend on the cellular ratio of NO/O_2_^- ^and the existence of growth factors in the surrounding tissues. The high EPR signal intensity resulting from the cell membrane lipids' degradation product, lipoperoxide radicals, LOO-, and the increased level of IL-6 in Group 1 indicates a prevalence of oxidative stress in severe stroke patients. In conditions where NO is more prevalent than superoxide anion (O_2_-), NO toxicity in neurons is decreased by restoration of peroxinitrite (ONOO-) to NO_2 _[25,26]. The increased concentrations of NO_2 _in Group 2 patients and the relatively diminished EPR signals of LOO- indicate conditions in which NO can act as an antioxidant. The protective response of NO can also be obtained through nitrosonium (NO^+^), which nitrosylates the thiol groups of glutamate receptors and thus diminishes glutamate toxicity [27].

The limitation of this study is that we lack a comprehensive understanding of the complex action of NO in the blood and CSF in the acute stage of ischemic stroke. The initial endothelial NO expressed in the blood might exhibit protective qualities, which is consistent with its ability to improve microhaemorheology [28]. Additionally, whether the CSF cytokine levels are dependent upon serum/blood concentrations and the blood-CSF barrier function and whether the CSF markers are synthesized purely intrathecally has yet to be evaluated.

## Conclusions

The results of the present investigation demonstrate that nitrate (NO_2_) content in the CSF appears to reflect the severity of the oxidative stress reaction that develops in the ischemic neurovascular unit in the first hours of stroke and can predict functional outcome. CSF IL-6 content seems to be the most reliable prognostic indicator in the acute phase of ischemic stroke, with regard to the probability of infarct size, the clinical course of disease and the functional outcome of stroke at one month.

## Competing interests

The authors declare that they have no competing interests.

## Authors' contributions

MB designed the study, interpreted the data and drafted the manuscript. TS carried out the immunoassays and EPR analysis. RS participated in acquisition of data, interpretation and statistical analysis. NI conducted the additional statistics and interpreted the data. NB revised critically the intellectual content and gave the final approval for the given version of manuscript.

All authors read and approved the final manuscript.

## Authors' Information

MB Associated Professor of Neurology MD, PhD. Department of Neuromedicine, Tbilisi State Medical University, Tbilisi, Georgia.

TS Full Professor of Biophysics PhD. Department of Biophysics and Biochemistry, Tbilisi State Medical University, Tbilisi, Georgia

RS Full Professor of Neurology MD, PhD. Department of Neuromedicine, Tbilisi State Medical University, Tbilisi, Georgia.

NI Associated Professor of Biophysics, PhD, Head of Medical Statistics Section of the Department of Biophysics and Biochemistry, Tbilisi State Medical University, Tbilisi, Georgia

NB Full Professor of Neurology, MD. Department Head, Tel-Aviv Sourasky Medical Center, Sackler Faculty of Medicine, Tel-Aviv University, Tel-Aviv, Israel

## Pre-publication history

The pre-publication history for this paper can be accessed here:

http://www.biomedcentral.com/1471-2377/11/41/prepub
